# Wandering spleen with splenic arteriovenous torsion

**DOI:** 10.1002/ccr3.5051

**Published:** 2021-11-07

**Authors:** Genichi Hashiguchi, Takashi Hamada, Mampei Yamashita, Tamotsu Kuroki

**Affiliations:** ^1^ Department of Surgery National Hospital Organization Nagasaki Medical Center Omura City Nagasaki Japan

**Keywords:** splenectomy, splenic arteriovenous torsion, splenomegaly, wandering spleen

## Abstract

Splenic arteriovenous torsion causes splenomegaly and ischemic necrosis of the spleen. The recommended treatment for wandering spleen with hypersplenism is considered to be splenectomy.

## Graphical abstract

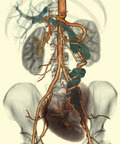

## 1 CASE PRESENTATION

A wandering spleen is a rare condition due to either the absence or laxity of the splenic ligaments. Splenic arteriovenous torsion causes splenomegaly and ischemic necrosis of the spleen. The recommended treatment for wandering spleen with hypersplenism is considered to be splenectomy.

A 35‐year‐old Japanese woman had been diagnosed with a wandering spleen 20 years ago. No symptoms such as abdominal pain were observed before the delivery of her full‐term infant. Contrast‐enhanced computed tomography (CE‐CT) was conducted post‐delivery. The patient's spleen was located in the middle of the abdomen (A). A few months post‐delivery, she began to have frequent left‐sided abdominal pain. CE‐CT showed that her spleen had enlarged and moved into the pelvis (B). There is no dislocation of pancreas. The splenic artery and vein were spirally twisted 1260° (C). A laparoscopic splenectomy was performed (D). No abdominal pain occurred post‐surgery. The weight of the removed spleen was 350 g.

**FIGURE 1:**
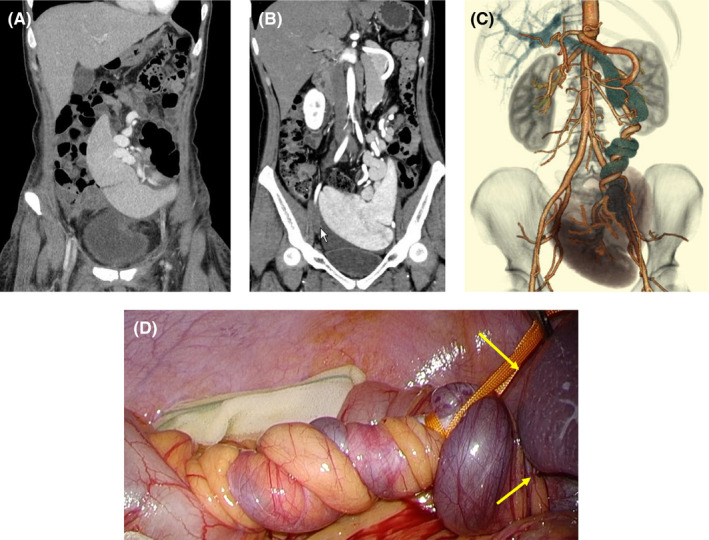
(A) CE‐CT after delivery. The spleen was located in the middle of the abdomen. (B) CE‐CT a few months post‐delivery. The spleen had become enlarged and it had also moved into the pelvis. (C) 3D construct CT. The splenic artery and vein were spirally twisted by 1260°. (D) Laparoscopic image. Yellow arrows (→) indicate the spleen. The splenic artery and vein were both spirally twisted

This was a rare case of wandering spleen with splenomegaly and abdominal pain due to splenic arteriovenous torsion after childbirth. Wandering spleen which moves from the left hypochondrium to other places in the abdominal cavity had been caused by the absence or laxity of splenorenal and gastrosplenic ligaments.^1^ Congestion of the splenic vein had thus caused splenomegaly and hypersplenism.^1^, ^2^ Abdominal pain developed because of an intermittent blood flow disturbance due to splenic arteriovenous torsion. In wandering spleen with hypersplenism, splenectomy is recommended instead of splenopexy.^2^

## CONFLICT OF INTEREST

The authors declare that there is no conflict of interest that could be perceived as prejudicing the impartiality of the research reported herein.

## CONSENT

Informed consent for publication and related images has been obtained from the patient.

## Data Availability

The data that support the findings of this study are available on request from the corresponding author. The data are not publicly available due to privacy or ethical restrictions.
